# Evaluation of Acute Pancreatitis Severity and Prognosis Using the Aggregate Systemic Inflammation Index (AISI) as a New Marker: A Comparison with Other Inflammatory Indices

**DOI:** 10.3390/jcm14103419

**Published:** 2025-05-14

**Authors:** Oğuzhan Zengin, Burak Göre, Oğuz Öztürk, Arap Merve Cengiz, Senanur Güler Kadıoğlu, Emra Asfuroğlu Kalkan, İhsan Ateş

**Affiliations:** 1 Department of Internal Medicine, Ankara Bilkent City Hospital, Ankara 06800, Turkey; mrvcngz95@gmail.com (A.M.C.); senanurgulerr@gmail.com (S.G.K.); emra.kalkan@hotmail.com (E.A.K.); dr.ihsanates@hotmail.com (İ.A.); 2 Department of Internal Medicine, Çerkeş State Hospital, Çankırı 18600, Turkey; goreburak1@gmail.com; 3 Department of Gastroenterology, Ankara Bilkent City Hospital, Ankara 06800, Turkey; oguzozturk90@gmail.com

**Keywords:** pancreatitis, biomarkers, inflammation, inflammatory markers, severity of illness index

## Abstract

**Background/Objectives:** Acute pancreatitis (AP) remains a pressing clinical challenge, largely due to its potential to lead to life-threatening complications and increased mortality. Over the years, numerous tools have been proposed to evaluate the intensity of AP and estimate likely health outcomes. Despite their usefulness, many of these assessment models are complex and rely on a wide array of clinical inputs, making them less practical in everyday healthcare settings. In contrast, the Aggregate Systemic Inflammation Index (AISI), which is calculated using routine blood count parameters, provides a simpler and more inclusive approach to measuring systemic inflammation. This research focuses on examining how effectively AISI can be used to gauge disease severity and project clinical trajectories in individuals affected by pancreatitis. **Methods:** This retrospective study reviewed the medical records of 412 individuals diagnosed with acute pancreatitis, all of whom received care at the Internal Medicine Clinic of Ankara Bilkent City Hospital between 1 April 2019 and 1 September 2024. The investigation encompassed a thorough analysis of patients’ demographic characteristics, lab parameters, and clinical findings, with special attention given to inflammatory markers, including the Aggregate Systemic Inflammation Index (AISI), its revised version, the Platelet-to-Lymphocyte Ratio (PLR), the Neutrophil-to-Lymphocyte Ratio (NLR), and the Systemic Inflammatory Response Index (SIRI). Comparative analyses between groups were performed using independent sample *t*-tests and one-way ANOVA, complemented by Tukey’s post hoc tests where appropriate. Correlations among continuous variables were determined through Pearson’s analysis, and the prognostic accuracy of both AISI and its modified form was assessed using Receiver Operating Characteristic (ROC) curve methodology. **Results:** The mean age among participants was 63.47 ± 17.92 years, while the average AISI value was calculated as 1183.89 ± 1067.42. Both the original and modified versions of the AISI index showed strong positive correlations with several key clinical measures, including prolonged hospitalization, a Glasgow score of 2 or above, BISAP, Ranson scoring, the revised Atlanta classification, and APACHE II. AISI was also significantly linked to the presence of complications and overall mortality (*p* < 0.01). Analysis through ROC curves demonstrated that an AISI level above 236.626 effectively predicted hospital stays exceeding 10 days, with a sensitivity of 94.40% and a specificity of 91.00%. Moreover, both AISI and its modified form reliably distinguished patients who had a Ranson score of zero, with high diagnostic accuracy. **Conclusions:** AISI and its modified version demonstrate a strong association with both the intensity and clinical course of acute pancreatitis. Thanks to their simplicity, low cost, and broad usability in healthcare settings, these indices hold considerable promise as practical and dependable tools for assessing the severity and likely outcomes of this increasingly prevalent disease.

## 1. Introduction

Acute pancreatitis (AP) is defined as an acute inflammatory condition of the pancreas, which can lead to systemic complications and significant morbidity. In severe cases, AP may result in multiple organ failure, which in turn prolongs hospital stay and increases mortality rates [1,2]. Therefore, accurately assessing the severity of the disease in the early stages is crucial for clinicians in guiding patient management and improving outcomes. Although several prognostic scoring systems, such as Ranson, modified Glasgow, and APACHE II, are available for AP, many of these rely on laboratory parameters that may not be readily available at the time of admission, or they require complex calculations, limiting their practical application. This presents particular challenges in emergency settings, where rapid decision making is essential [3,4,5,6]. Although the BISAP score is considered a relatively practical alternative, its dependence on radiological and certain laboratory data also restricts its broader applicability. These limitations highlight the growing need for new markers that are easily accessible, rapid, and reliable.

In recent years, inflammatory indices derived from complete blood count (CBC) parameters have gained attention for assessing systemic inflammation in AP and other conditions. Specifically, the neutrophil-to-lymphocyte ratio (NLR) and platelet-to-lymphocyte ratio (PLR) have emerged in the literature as simple yet effective indicators of immune response [7]. These ratios are thought to reflect the balance between neutrophil-mediated inflammation and lymphocyte-driven immune regulation [7]. In this context, the Aggregate Index of Systemic Inflammation (AISI), which has recently been proposed as a more comprehensive marker of systemic inflammation, aims to represent the overall inflammatory burden by incorporating neutrophil, monocyte, platelet, and lymphocyte counts into a single measure. Although many inflammatory indices have been investigated in acute pancreatitis, no studies have been identified in the literature specifically focusing on AISI. This gap necessitates an evaluation of its potential prognostic role in AP, where inflammation-related indices have already shown associations with disease outcomes.

While AISI has not yet been evaluated in the context of acute pancreatitis, it has shown prognostic value in various other clinical conditions such as hypertension, idiopathic pulmonary fibrosis, and periprosthetic joint infections. The inclusion of platelet count in its formula, compared to the SIRI index, may allow AISI to represent a broader inflammatory response. Platelets have been shown to play roles in directly recognizing, isolating, and eliminating pathogens, in recruiting and activating leukocytes at sites of infection and inflammation, and in modulating leukocyte behavior to enhance phagocytosis and pathogen clearance. Therefore, the presence of platelets in the AISI formula may enhance its ability to reflect systemic inflammatory responses more accurately [8,9,10,11,12]. Since local complications in AP typically take time to become radiologically apparent, the importance of accessible and reliable biomarkers to guide early clinical decisions is increasing. Although biomarkers such as CRP, procalcitonin, and LDH have demonstrated usefulness in predicting pancreatic necrosis, none has yet been clearly established as an ideal early-phase marker [13,14,15,16,17]. Furthermore, the limited routine availability of these tests across healthcare facilities also restricts their widespread use. In this study, the potential role of AISI and modified AISI in predicting disease severity and prognosis in patients with acute pancreatitis was investigated, with the additional aim of comparing these indices to other inflammatory markers such as NLR, PLR, and SIRI.

## 2. Methods

### 2.1. Study Design and Patient Selection

This retrospective, single-center study was conducted between 1 April 2019 and 1 September 2024 at Ankara Bilkent City Hospital. A total of 412 patients diagnosed with AP were included in the study. The study was approved by the Ankara Bilkent City Hospital Ethics Committee, and all procedures adhered to the ethical guidelines of the Declaration of Helsinki and its later revisions (Date: 2 October 2024, Decision No: TABED 2-24-542).

**Figure 1:**
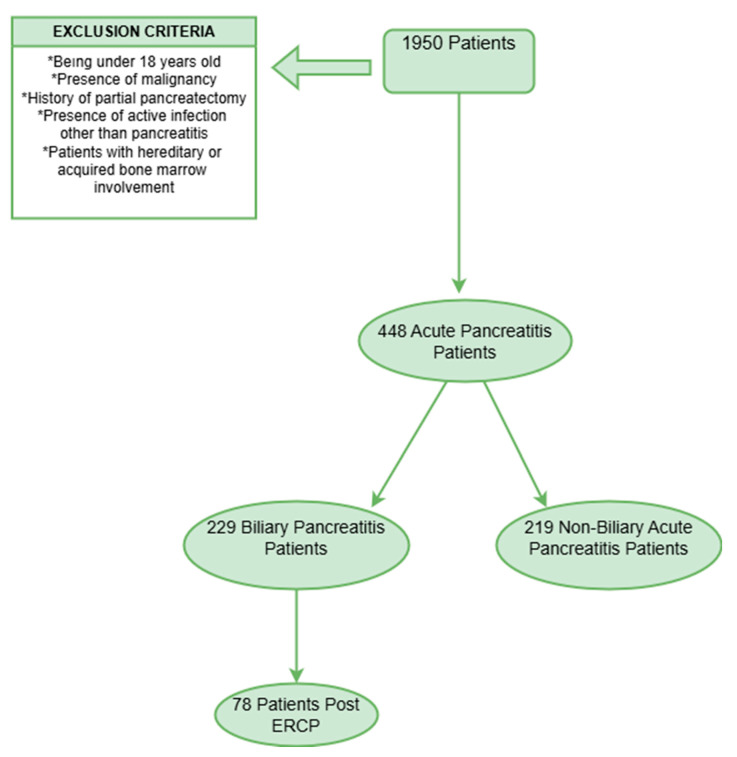
Flowchart of inclusion and exclusion criteria.

The inclusion criteria for the study were as follows: patients aged 18 and older and who presented to Ankara Bilkent City Hospital with a diagnosis of AP between 1 April 2019 and 1 September 2024. The criteria for diagnosing AP were defined as the presence of at least two of the following three features: prolonged abdominal pain specific to AP; serum amylase and/or lipase levels elevated up to three times the normal range; characteristic findings of AP on magnetic resonance imaging (MRI) or computed tomography (CT) scans. The local complications were identified as necrosis, peripancreatic fluid accumulation, pseudocyst, and pancreatic ascites, while the systemic complications included respiratory, cardiac, and renal insufficiencies, acute respiratory distress syndrome, sepsis, and multiple organ failure [18]. The exclusion criteria were as follows: patients under the age of 18, patients diagnosed with malignancy, patients with a history of partial pancreatectomy, patients with active infections unrelated to pancreatitis, patients with hereditary or acquired bone marrow involvement (such as Immune Thrombocytopenic Purpura, Aplastic Anemia, Chronic Lymphocytic Leukemia, Chronic Myeloid Leukemia, Myelodysplastic Syndrome, etc.), and patients with missing data on the main parameters. Inclusion and exclusion criteria are illustrated in Figure 1.

### 2.2. Data Collection

For each patient, age, gender, and clinical and laboratory data were evaluated. During hospitalization, laboratory parameters such as complete blood count and biochemical markers were examined. In patients for whom disease severity and clinical evaluation were feasible, Glasgow, APACHE II, Ranson, Marshall, BISAP, HAPS, and Balthazar scores were calculated, in addition to the Atlanta classification.

### 2.3. Inflammatory Index Calculations

The Neutrophil-to-Lymphocyte Ratio (NLR) was calculated by dividing the absolute neutrophil count by the absolute lymphocyte count (NLR = Neutrophil count/Lymphocyte count); the Platelet-to-Lymphocyte Ratio (PLR) was calculated by dividing the absolute platelet count by the absolute lymphocyte count (PLR = Platelet count/Lymphocyte count); the Lymphocyte-to-Monocyte Ratio (LMR) was calculated by dividing the absolute lymphocyte count by the absolute monocyte count (LMR = Lymphocyte count/Monocyte count). The Neutrophil-to-Lymphocyte*Platelet Ratio (NLRP) was calculated using the formula (NLRP = [Neutrophil count/Lymphocyte count] × Platelet count). The Systemic Inflammatory Response Index (SIRI) was calculated using the formula (SIRI = Neutrophil count × Monocyte count/Lymphocyte count). The BISAP score was assessed on the day of admission. AISI was calculated using the following formula: (Neutrophil count × Platelet count × Monocyte count)/Lymphocyte count. The Modified AISI score was determined using the following formula: (1 + (Age > 70) + (LDH > 350) + (AST > 250)) × AISI. In this formula: If the age parameter is greater than 70, a value of 1 is assigned; otherwise, a value of 0 is given. If the LDH value is above 350, a value of 1 is assigned; otherwise, 0 is given. If the AST (Aspartate Aminotransferase) value is above 250, a value of 1 is assigned; otherwise, 0 is given. In this way, each parameter receives a value of 1 if it exceeds the specific threshold and 0 if it is below. Finally, these obtained values are multiplied by the AISI score. The Glasgow 2 score was calculated based on eight clinical and laboratory parameters assessed within the first 48 h of admission, with a total score ranging from 0 to 8. The Marshall score was used to evaluate organ dysfunction across three systems (respiratory, renal, and cardiovascular), with scores ranging from 0 to 12. The BISAP score was assessed on the day of admission using five clinical variables and ranged from 0 to 5. The Ranson score was determined using eleven parameters, five of which were evaluated at admission and six at 48 h, yielding a total score between 0 and 11. The APACHE II score was calculated based on physiological measurements, age, and chronic health conditions, with scores ranging from 0 to 71. The HAPS score was evaluated using three clinical criteria; a score of 0 indicates a low-risk course, whereas scores between 1 and 3 indicate a non-benign course. According to the 2021 revision of the Atlanta classification, the severity of acute pancreatitis is divided into three categories: mild, moderately severe, and severe. Mild acute pancreatitis is characterized by the absence of organ failure and local or systemic complications. Moderately severe acute pancreatitis involves transient organ failure, which resolves within 48 h, and/or the presence of local or systemic complications. In contrast, severe acute pancreatitis is defined by persistent organ failure lasting longer than 48 h.

### 2.4. ERCP Procedure

Endoscopic retrograde cholangiopancreatography (ERCP) procedures in this study were carried out at Ankara Bilkent City Hospital by gastroenterologists with substantial experience in the field. All procedures followed established clinical protocols that were in line with the recommendations from both the American Society for Gastrointestinal Endoscopy (ASGE, 2020) and the European Society of Gastrointestinal Endoscopy (ESGE, 2019). Indications for ERCP were determined based on a comprehensive assessment that included the patient’s clinical presentation, relevant laboratory values (particularly, elevated cholestatic enzymes and bilirubin), and imaging findings, all evaluated collaboratively by a multidisciplinary team. Prior to the procedure, each patient received detailed information about the purpose of the intervention, potential risks, and possible complications. Written informed consent was obtained from all participants. The procedures were performed under sedation with fluoroscopic guidance. In this particular cohort, no patient required pancreatic duct intervention, and as such, there were no cases involving pancreatic duct cannulation or stent placement. All interventions were focused on the biliary tract, with endoscopic sphincterotomy and/or stone extraction performed where necessary. Furthermore, no prophylactic antibiotics or pharmacologic pre-treatment was administered to any of the patients.

### 2.5. Statistical Analysis

In this study, the obtained data were analyzed using the SPSS (Statistical Package for the Social Sciences) software for statistical analysis (SPSS version 26.0 IBM Corp., Armonk, NY, USA). The Shapiro–Wilk test was employed to evaluate the normality of the data. Parametric tests were conducted for variables with a normal distribution, whereas non-parametric tests were applied to those without a normal distribution. Demographic data and clinical measurements were summarized using descriptive statistics. Mean and standard deviation were used to describe the distribution of continuous variables, while frequency distributions and percentages were calculated for categorical data. Relationships between variables were examined using the Pearson Correlation Test and Spearman Correlation Test. Correlation analyses were conducted to determine the relationships between inflammatory markers (AISI, Modified AISI, PLR, SIRI, NLRP, NLR) and the clinical parameters of patients (hospitalization duration, Glasgow 2 score, BISAP score, Atlanta classification, Ranson score, APACHE II score, etc.). For comparing differences between groups, the independent sample *t*-test and ANOVA were used as parametric tests. In cases where the data did not follow a normal distribution, the Mann–Whitney U test and Kruskal–Wallis test were applied. Specifically, the relationships between hospitalization duration and clinical parameters were examined, and the associations between AISI and Modified AISI with these parameters were analyzed. ROC analysis was performed to examine the relationship between AISI and Modified AISI with key clinical outcomes, such as hospitalization duration, mortality, and complications. The analysis calculated the sensitivity, specificity, positive predictive value (PPV), and negative predictive value (NPV) for each inflammatory marker. These tests were then evaluated for their clinical relevance. Due to the retrospective nature of the study, hemogram data could not be obtained in a consistent and time-standardized manner for all patients. The timing of laboratory tests varied across patients, and in some cases, certain tests were not requested at all. Additionally, clinical factors such as the patients’ hydration status, the duration of oral intake restriction, and differences in medical treatments administered during follow-up introduced further variability. These inconsistencies significantly limited the reliability and comparability of serial AISI measurements. Therefore, only the AISI values at the time of hospital admission were included in the analysis. Although CRP, procalcitonin, and IL-6 are well-established inflammatory markers, they were not analyzed in this study because these parameters are not routinely requested in our clinic for acute pancreatitis, and the retrospective dataset lacked sufficient data for these variables.

A power analysis was conducted using G*Power software (version 3.1.9.7) to determine whether the study had an adequate sample size. In the analysis, a 95% confidence level and 80% test power were applied, and it was determined that at least 130 participants were needed for the study. Statistical significance was defined as *p* < 0.05.

## 3. Results

The demographic profile of the participants and the distribution of various clinical measurements are shown in Table 1. The average age of the participants was 63.47 ± 17.92 years, with an age range from 23 to 98 years. The average hospitalization duration was 10.60 ± 8.75 days, with a range from 0 to 64 days. The mean AISI score was 1183.89 ± 1067.42, and the mean Modified AISI score was 2075.35 ± 2629.51. Other clinical parameters included a mean Glasgow 2 score of 1.12 ± 1.08, a mean Marshall score of 0.28 ± 0.98, a mean BISAP score of 0.99 ± 1.08, a mean Ranson score of 1.89 ± 1.42, a mean APACHE II score of 5.67 ± 4.25, and a mean HAPS score of 0.65 ± 0.63. Regarding gender distribution, 53.16% of the participants were female and 46.84% were male. The proportion of patients with biliary disease was 51.46%, and 58.01% of patients did not undergo ERCP. The rate of systemic complications was 29.13%, and the rate of local complications was 19.90%. The proportion of patients with post-ERCP secondary complications was 17.96%, while the mortality rate was 3.64%.

Table 2 presents the relationships between the blood parameter measurements and clinical evaluation criteria of the patients. The analyses reveal that inflammation markers, particularly AISI and Modified AISI scores, exhibit significant correlations with clinical parameters. It was found that PLR, NLRP, NLR, AISI, and Modified AISI exhibited positive correlations with the patients’ hospitalization duration, Glasgow 2 score, BISAP, Ranson, APACHE II, and HAPS scores (*p* < 0.01). These relationships highlight the importance of inflammation in determining the severity of pancreatitis. Furthermore, strong correlations were found between these markers and systemic complications, with significant positive relationships detected in both indices (AISI and Modified AISI) (*p* = 0.001). Other analyses in Table 2 revealed that AISI and Modified AISI showed weaker correlations with mortality rates, with *p*-values of 0.013 and 0.145, respectively. However, it was found that AISI could detect the likelihood of patients staying in the hospital for 10 days or more with high sensitivity and specificity (94.40% and 91.00%, respectively). These findings suggest that AISI could be used as an important biomarker for predicting the severity of pancreatitis and improving clinical management. While Table 2 provides a summary of the distribution of laboratory parameters, their relationships with clinical severity scores and outcomes are further analyzed in Table 3, Table 4 and Table 5.

Table 3 presents the results of Pearson correlation analyses for continuous variables (age and length of hospital stay) and independent sample *t*-tests for binary clinical variables (e.g., systemic complications, mortality, sex). Correlation coefficients (r), t statistics (t), and associated *p*-values are reported. A *p*-value less than 0.05 was considered statistically significant and is marked with an asterisk (*). Detailed definitions of clinical variables are provided in the Methods section. Abbreviations and criteria for statistical significance are explained in the footnote below the table. The findings indicate that longer hospital stays were significantly associated with higher levels of BUN (r = 0.237, *p* < 0.001), CRP (r = 0.212, *p* < 0.001), and direct bilirubin (r = 0.187, *p* < 0.001). In patients with systemic complications, levels of CRP (t = −5.023, *p* < 0.001), BUN (t = −3.944, *p* < 0.001), and LDH (t = −2.592, *p* = 0.001) were significantly elevated. Regarding mortality, ALT (t = −5.471, *p* < 0.001), AST (t = −3.328, *p* < 0.001), and CRP levels showed significant differences. In cases where post-ERCP pancreatitis developed, increased levels of amylase (t = 3.692, *p* < 0.001), LDH (t = 3.893, *p* < 0.001), leukocytes (t = 4.013, *p* < 0.001), and neutrophils (t = 3.826, *p* < 0.001) were observed. When evaluating sex differences, male patients exhibited significantly lower levels of alkaline phosphatase (t = −5.618, *p* < 0.001) and direct bilirubin (t = −5.001, *p* < 0.001). Additionally, although correlations between age and laboratory parameters were generally weak, they were statistically significant in several cases, including BUN (r = 0.386, *p* < 0.001), creatinine (r = 0.179, *p* = 0.001), and hemoglobin (r = 0.247, *p* < 0.001).

The correlations between laboratory parameters and clinical severity scores in patients with acute pancreatitis are presented in Table 4. Among all variables, blood urea nitrogen (BUN) showed the strongest positive correlations with the APACHE II (r = 0.625, *p* < 0.001), BISAP (r = 0.546, *p* < 0.001), and Marshall (r = 0.480, *p* < 0.001) scores. C-reactive protein (CRP) was also significantly correlated with BISAP (r = 0.349, *p* < 0.001), APACHE II (r = 0.301, *p* < 0.001), and Glasgow 2 (r = 0.327, *p* < 0.001) scores, highlighting its role in systemic inflammation. Furthermore, serum creatinine levels were strongly associated with both Marshall (r = 0.611, *p* < 0.001) and APACHE II (r = 0.530, *p* < 0.001) scores. Lactate dehydrogenase (LDH) showed a particularly strong correlation with Ranson (r = 0.406, *p* < 0.001) and Marshall (r = 0.298, *p* < 0.001) scores. Leukocyte count demonstrated significant positive correlations with Ranson (r = 0.262, *p* < 0.001) and BISAP (r = 0.179, *p* < 0.001) scores, while neutrophil count was positively correlated with Glasgow 2 (r = 0.284, *p* < 0.001), Ranson (r = 0.295, *p* < 0.001), and APACHE II (r = 0.206, *p* < 0.001) scores. Conversely, lymphocyte count showed significant negative correlations with Ranson (r = 0.277, *p* < 0.001) and BISAP (r = 0.241, *p* < 0.001) scores, reflecting immune suppression in severe cases. In addition, hemoglobin levels were moderately correlated with APACHE II (r = 0.314, *p* < 0.001) and HAPS (r = 0.324, *p* < 0.001) scores. While the amylase levels exhibited weak to moderate correlations with certain scores (e.g., BISAP and Ranson), their overall correlation coefficients were relatively lower. Liver function markers such as total bilirubin, direct bilirubin, GGT, AST, and ALT exhibited weaker and more inconsistent associations across scoring systems. Albumin levels, however, showed a statistically significant correlation with APACHE II (r = 0.135, *p* = 0.006) and HAPS (r = 0.139, *p* = 0.005) scores. These findings underscore the prognostic value of systemic inflammation and organ function markers—particularly BUN, CRP, creatinine, LDH, leukocyte, neutrophil, and hemoglobin—in assessing the severity of acute pancreatitis.

Table 5 illustrates how different inflammation-related blood values relate to clinical indicators and outcomes in patients diagnosed with acute pancreatitis. For continuous variables such as hospitalization duration or age, correlation analyses were performed using Pearson’s test. In contrast, binary outcomes like mortality or the presence of complications were compared using independent sample *t*-tests. Markers such as AISI and SIRI stood out with their consistent associations across multiple clinical severity scores. For instance, both AISI (r = 0.220, *p* < 0.001) and SIRI (r = 0.239, *p* < 0.001) were moderately linked with hospital stay duration. AISI also showed positive relationships with several established severity scoring systems, including Glasgow ≥2, BISAP, Ranson, and APACHE II (all *p* < 0.001). In particular, the modified version of AISI yielded a notably high correlation with the Ranson score (r = 0.503, *p* < 0.001), implying a closer link with more severe clinical presentations. SIRI also followed a similar trend and was correlated with all severity metrics examined, including Glasgow and Ranson scores. Additionally, NLR, PLR, and NLRP were statistically associated with BISAP and Glasgow classifications, reinforcing the broader trend of inflammation-based parameters aligning with more severe disease. Group comparisons revealed that patients who experienced systemic complications had significantly higher values across several markers, including AISI, SIRI, and Modified AISI (*p* < 0.001 in each case). Furthermore, both AISI and SIRI differed significantly in individuals who developed local complications (*p* = 0.025 and *p* = 0.047, respectively). Regarding mortality, elevated NLR and SIRI levels were linked with fatal outcomes (*p* = 0.017 and *p* = 0.043). Notably, patients who developed post-ERCP pancreatitis had significantly increased values of SIRI and Modified AISI, indicating a potential predictive signal (*p* < 0.001). Altogether, these findings support the relevance of readily available blood count–based indices—particularly those incorporating neutrophils, lymphocytes, monocytes, and platelets—as useful tools for identifying clinical severity and predicting unfavorable events in patients hospitalized with acute pancreatitis.

Table 6 presents a comparison of systemic inflammation indices across three severity levels of acute pancreatitis, based on the revised Atlanta classification. The analysis revealed a stepwise increase in the mean values of all measured indices as disease severity progressed from mild to moderate and severe. All differences were statistically significant according to one-way ANOVA (*p* < 0.001 for each index). The mean PLR increased from 236.91 ± 125.70 in the mild group to 273.38 ± 156.11 in the moderate group, and reached 354.90 ± 178.20 in the severe group. A similar trend was observed for MLR, with mean values of 0.43 ± 0.24, 0.52 ± 0.27, and 0.66 ± 0.35 across the three groups, respectively. The increase in NLR was particularly pronounced, with group means of 7.29 ± 5.59 in mild, 11.86 ± 10.23 in moderate, and 15.17 ± 11.58 in severe pancreatitis. Among the more comprehensive indices, AISI demonstrated a notable increase from 920.21 ± 816.70 in the mild group to 1382.59 ± 1055.16 in the moderate group, and further to 2064.64 ± 1641.24 in the severe group. The Modified AISI values rose even more sharply, from 1538.16 ± 2034.23 to 2354.62 ± 2352.49 and then to 4261.11 ± 4573.60. Lastly, the SIRI showed consistent escalation, with mean values of 3.51 ± 3.00, 5.53 ± 4.34, and 7.97 ± 7.07 in the mild, moderate, and severe groups, respectively. Post hoc analyses using Tukey’s test confirmed that each index differed significantly between the three groups (*p* < 0.05), following a consistent pattern of severe > moderate > mild. This suggests that these inflammatory indices reliably reflect the progression of clinical severity in acute pancreatitis. Taken together, these results underscore the close link between systemic inflammation and disease severity and support the potential use of these indices—particularly NLR, AISI, and Modified AISI—as accessible and informative tools for early risk stratification and clinical decision making.

**Figure 2:**
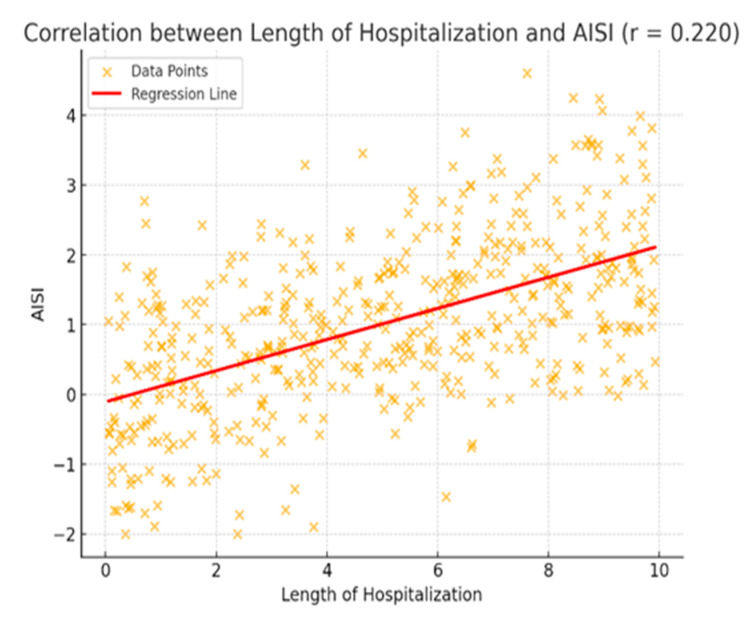
Scatter plot demonstrating the correlation between hospitalization duration and AISI scores. Each orange dot represents an individual patient. The red line illustrates the linear regression trend.

A positive correlation was found between the length of hospitalization and AISI (r = 0.220). As shown in Figure 2, AISI values tend to increase as the duration of hospitalization increases. The red regression line fitted among the data points visually supports this trend. This finding suggests that patients with longer hospital stays may experience higher levels of inflammatory stress. However, the relatively low correlation coefficient indicates that the strength of this association is weak and that other factors may also influence AISI levels.

**Figure 3:**
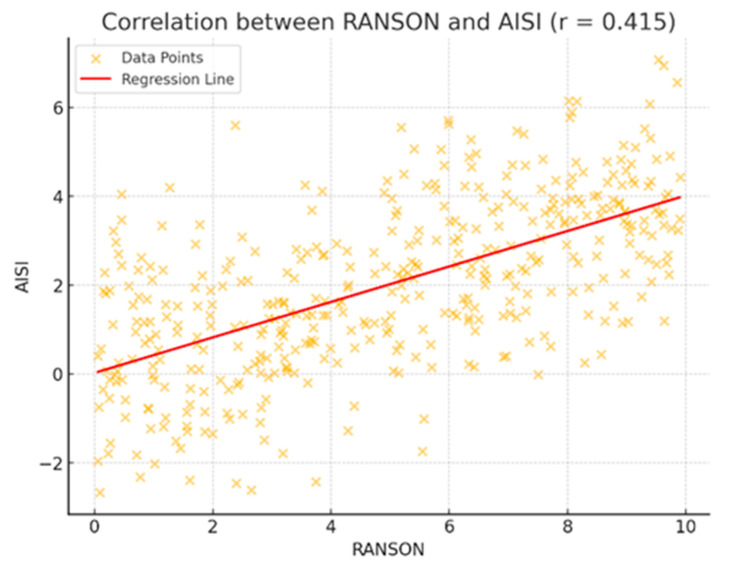
Scatter plot showing the correlation between RANSON score and AISI (r = 0.415). Each orange point represents an individual patient. The red line indicates the linear regression trend.

A moderate positive correlation was observed between the RANSON score and AISI (r = 0.415). As shown in Figure 3, higher RANSON scores were generally associated with increased AISI values. The distribution of data points and the fitted linear regression line (red) support the presence of a meaningful linear relationship between the two variables. This finding suggests that patients with more severe clinical presentations, as indicated by higher RANSON scores, may also exhibit elevated inflammatory responses.

**Figure 4:**
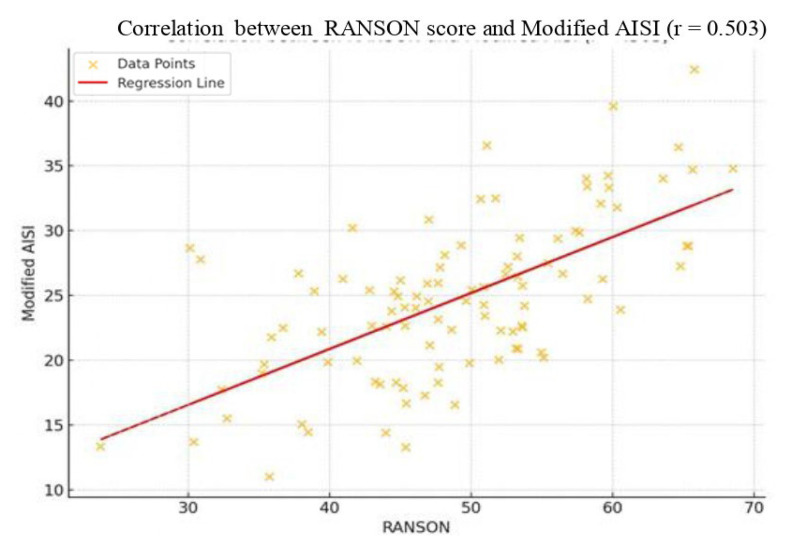
Scatter plot showing the correlation between RANSON score and Modified AISI (r = 0.503). Each orange point represents an individual patient. The dashed red line indicates the linear regression trend.

A strong positive correlation was identified between the RANSON score and Modified AISI (r = 0.503). As illustrated in Figure 4, higher RANSON scores were associated with elevated Modified AISI values. The linear regression line (dashed red) clearly demonstrates a consistent upward trend across the dataset, indicating a stronger relationship compared to the original AISI.

The ROC analysis results presented in Table 7 show the values calculated based on the most likely method for AISI and Modified AISI levels according to the designated reference categories of the variables.

Hospitalization Duration: The reference category was taken as under 10 days. For the over 10 days category, the ROC value was calculated as 236.626, and the (*) symbol indicates that this value is statistically significant according to the AISI level.

Ranson Status: The 0 status was taken as the reference category. The ROC value for the 0 category was calculated as 113.189, and the (*) symbol indicates that this value is statistically significant according to the AISI level. Based on this finding, the AISI level in patients below this value indicates a Ranson score of 0.

**Figure 5:**
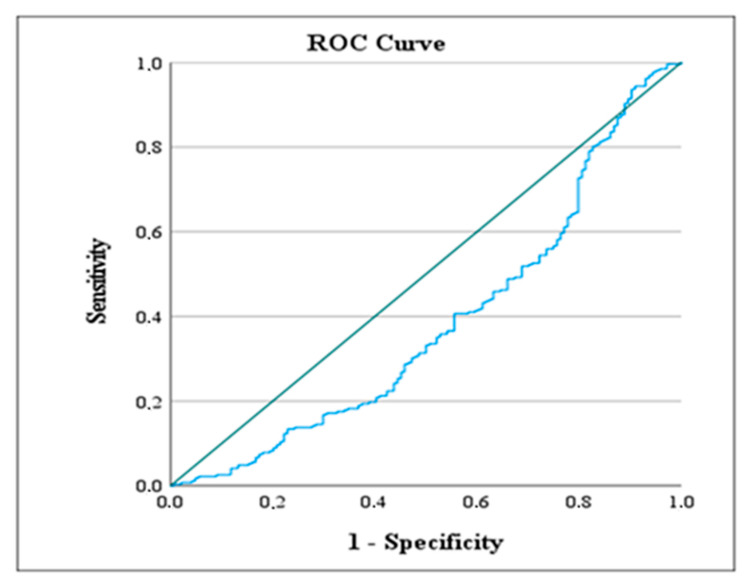
ROC curve demonstrating the predictive value of the AISI score for prolonged hospitalization (>10 days). The blue line represents the sensitivity and specificity trade-off at different threshold levels of AISI. It illustrates how the true positive rate (sensitivity) varies with the false positive rate (1—specificity) as the decision threshold changes.

**Figure 6:**
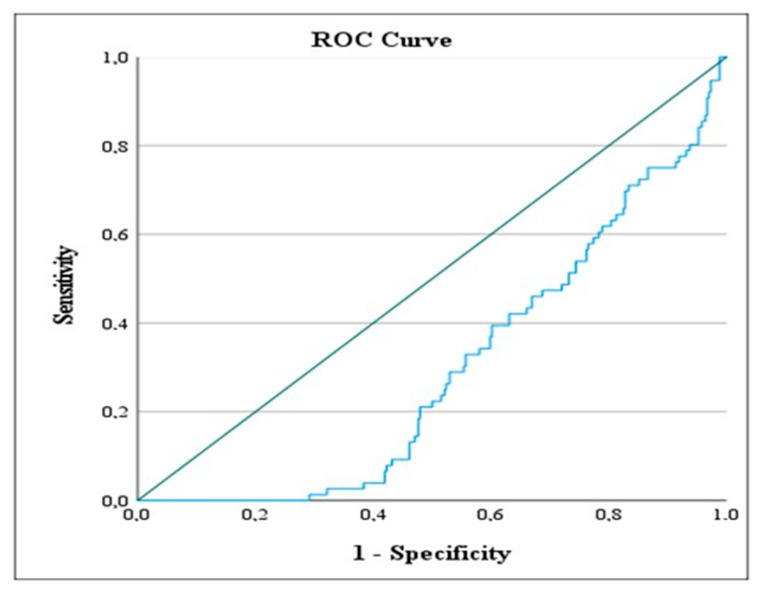
ROC curve depicting the predictive performance of AISI for identifying patients with a RANSON score of 0. The blue line represents the sensitivity and specificity trade-off at different threshold levels of AISI. It illustrates how the true positive rate (sensitivity) varies with the false positive rate (1—specificity) as the decision threshold changes.

**Figure 7:**
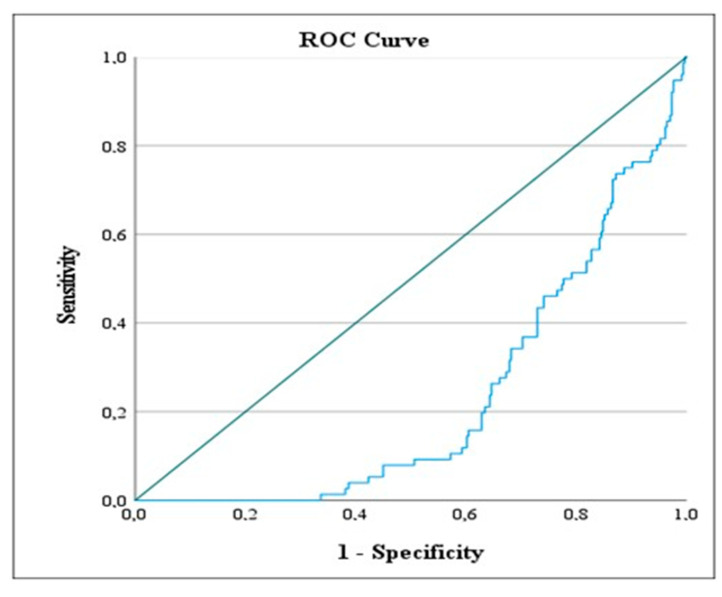
ROC curve illustrating the predictive accuracy of Modified AISI for patients with a RANSON score of 0. The blue line represents the sensitivity and specificity trade-off at different threshold levels of AISI. It illustrates how the true positive rate (sensitivity) varies with the false positive rate (1—specificity) as the decision threshold changes.

Ranson Status: The 0 status was taken as the reference category. The ROC value for the 0 category was calculated as 99.287, and the (**) symbol indicates that this value is statistically significant according to the Modified AISI level. Based on this finding, patients with a Modified AISI level below this value show a Ranson score of 0. The ROC analysis results are visually presented in Figure 5, Figure 6 and Figure 7. These figures illustrate the values calculated based on the designated reference categories of the variables according to AISI and Modified AISI levels. Figure 5 shows the ROC curve for hospitalization duration, Figure 6 details the Ranson score based on AISI levels, and Figure 7 presents the Ranson score based on Modified AISI levels.

## 4. Discussion

Acute pancreatitis is a common health issue worldwide and carries a high mortality risk [19]. Accurately determining the severity of pancreatitis is essential for planning the most appropriate treatment. Many assessment scores are based on various clinical evaluations and biochemical tests. These assessment scores require specific time frames and multiple parameters. However, the AISI score, due to its strong correlation with disease severity and simple calculation, provides a valuable scoring system for risk assessment in resource-limited settings. AISI, as a biomarker index that comprehensively reflects the severity of inflammation, differs from existing indices [20].

In our study, we examined the previously unexplored role of AISI in pancreatitis severity and prognosis in the literature. The results demonstrated the effectiveness of AISI in assessing disease severity and its strong relationship with clinical parameters, highlighting its clinical importance. Both AISI and Modified AISI were found to be valuable prognostic tools for assessing pancreatitis severity. AISI showed strong positive correlations with other severity indicators, including hospitalization duration, Glasgow 2 score, BISAP, Ranson, and APACHE II (*p* < 0.01). Additionally, both indices were strongly related to systemic complications, which may indicate the potential for identifying patients who will develop systemic complications. ROC analysis confirmed AISI’s predictive accuracy, showing excellent sensitivity and specificity. The AISI score reliably predicted the likelihood of patients requiring hospitalization for more than 10 days, with a sensitivity of 94.40% and a specificity of 91.00%. These results demonstrate AISI’s potential to accurately predict the prognosis of pancreatitis patients. Furthermore, AISI identified patients with a Ranson score of 0 with high accuracy, showing a sensitivity of 100% and a specificity of 98.80%. This is important in centers where biochemical parameters cannot be assessed, as it can demonstrate a low Ranson score using only hemogram values. In clinical practice, it is important to use a combination of biomarkers and indices when assessing risks and predicting outcomes for AP patients [21].

Senol et al. explored the role of RDW (Red Blood Cell Distribution Width) in their study and found that RDW levels at the time of hospitalization were significantly associated with mortality in AP [22]. WBC and neutrophil count can worsen the inflammatory response in AP. Additionally, in severe AP, platelets and white blood cells are activated, which can accelerate disease progression and the onset of complications [23]. Therefore, we believe that platelet and WBC values are important when calculating the inflammation index and should be included in these formulas. Based on these findings, we believe that AISI, which integrates various inflammatory markers, could contribute to a more comprehensive understanding of the pathophysiology of AP. Many factors play a role in predicting the progression of AP, and interleukins released from various cellular sources such as monocytes, macrophages, endothelial cells, and fibroblasts play a critical role in the inflammatory process [24,25,26]. WBC has been shown in previous studies to be an important marker for predicting the severity of AP [27]. In severe cases, pancreatitis can lead to the release of cytokines such as Interleukin-1, Interleukin-6, and Tumor Necrosis Factor-α, which activate neutrophils, monocytes, lymphocytes, and platelets, worsening the inflammatory response [28]. This situation demonstrates that inflammatory markers should encompass all these cellular responses when assessing the severity of pancreatitis. Considering all these markers will provide a more comprehensive and clear indicator in evaluating the severity of pancreatitis. Other serum markers such as C-reactive protein, procalcitonin, interleukin-6, and interleukin-8 have been shown to predict the severity of AP; however, their use is not easily applicable in clinical practice and has not been clearly validated [29]. In our study, C-reactive protein and procalcitonin levels were not routinely monitored in some patients, and since there is no clear evidence in the literature regarding the relationship between these parameters and pancreatitis, these markers were not our focus. AISI, by encompassing all parameters of inflammation and offering a broader perspective compared to other inflammatory indices, could make a significant contribution to the clinical management of inflammation-related diseases such as pancreatitis. Because the parameters it includes were previously present in scoring systems used for determining severity in the literature. The use of AISI, instead of more limited indices like NLR and PLR, can provide more comprehensive data during the treatment process, thereby helping to develop personalized treatment approaches. NLR is considered a strong biomarker for determining the severity of AP. Additionally, findings support that high NLR is consistent with an increased mortality risk associated with AP and other inflammatory diseases [30]. Similarly, in our study, a relationship with mortality was observed. High NLR is generally considered to reflect imbalances in the immune response, indicating that neutrophil-driven inflammation is more prominent than lymphocyte-mediated regulation, consistent with previous studies on systemic inflammatory diseases [31]. Kong et al., discussing the high sensitivity and specificity of NLR, have suggested that this biomarker could be integrated into initial evaluation protocols for AP, allowing for the classification of patients according to their risk levels without the need for urgent CT imaging or complex biochemical tests [32]. In a study by Junare et al., it was suggested that as inflammation progresses, the number of lymphocytes decreases, which further elevates the NLR value [33]. Cho et al. reported that PLR could only predict the severity of AP in biliary pancreatitis [34]. In contrast to this study, our research did not focus solely on biliary pancreatitis, which was a strength of our study. We found that the AISI score is a stronger prognostic indicator than PLR compared to all other scoring systems. SIRI is a commonly used parameter in patients with acute pancreatitis and is calculated using neutrophil, monocyte, and lymphocyte counts. AISI, on the other hand, incorporates platelet count in addition to these cellular parameters, offering the potential to reflect the inflammatory response in a more comprehensive manner [12]. In this study, both indices were found to be statistically significantly associated with clinical severity scores. However, the stronger correlation coefficients observed between AISI and scores such as Ranson and APACHE II suggest that AISI may serve as a more robust indicator for assessing disease severity in acute pancreatitis.

BISAP is a scoring system used to assess the severity of pancreatitis. This system offers an effective method to identify patients at high risk of death and those at risk of developing severe AP within 24 h, and it has been validated as a reliable risk classification tool [35]. However, the need for biochemical tests to calculate the score, the detection of pleural effusion, and the assessment of mental status in individuals with cognitive disorders present various challenges. Recently, in addition to inflammatory indices, new indices such as the Neutrophil-to-Creatinine Ratio have also been considered for evaluating the severity of AP [36]. The investigation of new indices may be significant for common diseases such as AP.

Complications, which are important prognostic indicators in the follow-up of AP, have been previously associated with various laboratory parameters in the literature. Biomarkers such as BISAP, PLR, NLR, LMR, complete blood count, BUN, creatinine, and RDW are associated with the severity of pancreatitis and play a significant role in predicting the development of organ failure. ROC analyses have shown that most of these biomarkers have significant prognostic value in predicting the development of pancreatic organ failure. However, no significant difference has been observed among the inflammation indicators assessed using BISAP [37]. Inflammatory biomarkers such as CRP, PCT, and LDH have been shown in the literature to play a role in evaluating the course and severity of AP. These biomarkers have also been associated with the development of pancreatic necrosis. However, in our study, we did not analyze CRP and PCT values, as they are not routinely measured in cases of acute pancreatitis at our hospital [9,38]. The relationship between NLR levels and complications of AP has been previously reported in the literature [39]. However, in our study, systemic and local complications were assessed separately. Similar to this study, we found a significant relationship between NLR levels and systemic complications, but we were unable to determine its relationship with local complications. On the other hand, we identified a relationship between the AISI score and both local and systemic complications.

In our study, a strong relationship between the AISI score and both systemic and local complications was identified. In conclusion, it is believed that the early determination of the severity of pancreatitis using the AISI score could play a significant role in the clinical management and personalized treatment strategies for patients. The findings obtained offer promising results for the integration of AISI into clinical practice and facilitate the more effective management of inflammatory diseases such as pancreatitis. We believe that an index based solely on hematological markers could serve as a guide for physicians in all stages of pancreatitis. Early identification of high-risk patients using the AISI score could enable interventions like timely therapeutic adjustments. However, for AISI to be fully validated in conditions like pancreatitis, larger-scale, multi-center studies are required. These inflammation markers offer considerable value in clinical settings due to their cost-effectiveness, practicality, and economic feasibility.

Like other retrospective studies, this research has its limitations. Being conducted at a single center naturally narrows how broadly the results can be applied to other patient groups with different backgrounds or clinical settings. The lack of long-term follow-up is another shortcoming, as it prevents us from fully understanding how patients fare over time. Additionally, some commonly used inflammatory markers—such as CRP, PCT, and IL-6—could not be evaluated due to insufficient data in the medical records. For more definitive conclusions, future studies should ideally be prospective in design, include more diverse populations, and consider a wider range of clinical and laboratory variables.

## 5. Conclusions

This study examined the role of the AISI and its modified version in evaluating the severity and predicting the outcomes of pancreatitis. The findings demonstrated a strong correlation between both AISI and Modified AISI scores with disease severity and the duration of hospitalization in patients with acute pancreatitis. ROC curve analysis revealed that an AISI score above 236.626 was closely associated with patients who required prolonged hospitalization, specifically more than 10 days. This threshold was highly predictive of extended hospital stays, achieving a sensitivity of 94.40% and specificity of 91.00%. Additionally, AISI scores were significantly correlated with the development of systemic complications. These results suggest that AISI could be an effective biomarker for determining the severity and forecasting the prognosis of pancreatitis.

## Figures and Tables

**Table 1:** Demographic, clinical, and severity score characteristics of patients with acute pancreatitis.

**A. Continuous Variables**			
**Variable**	**Mean ± SD (Median)**	**Min.–Max.**	
Age	63.47 ± 17.92 (62)	23–98	
Hospitalization Time (Days)	10.60 ± 8.75 (8)	0–64	
AISI	1183.89 ± 1067.42	61.53–6869.41	
Modified AISI	2075.35 ± 2629.51	83.77–20,608.24	
Glasgow 2	1.12 ± 1.08	0.00–5.00	
Marshall	0.28 ± 0.98	0.00–12.00	
BISAP	0.99 ± 1.08	0.00–5.00	
RANSON	1.89 ± 1.42	0.00–9.00	
APACHE II	5.67 ± 4.25	0.00–26.00	
HAPS	0.65 ± 0.63	0.00–2.00	
**B. Categorical Variables**			
**Variable Category**	**N (%)**		
Gender			
Female	219 (53.16)		
Male	193 (46.84)		
Biliary Disease			
No	200 (48.54)		
Yes	212 (51.46)		
ERCP History			
No	239 (58.01)		
Yes	173 (41.99)		
Systemic Complication			
No	292 (70.87)		
Yes	120 (29.13)		
Local Complication			
No	330 (80.10)		
Yes	82 (19.90)		
Post-ERCP Pancreatitis			
No	338 (82.04)		
Yes	74 (17.96)		
Mortality			
No	397 (96.36)		
Yes	15 (3.64)		
Atlanta	Mild	239.00	(58.01)
	Moderate	131.00	(31.80)
	Severe	4.00	(10.19)

Baseline demographic characteristics, clinical parameters, and severity scores of patients diagnosed with acute pancreatitis. Continuous variables are presented as mean ± standard deviation [median], along with their minimum and maximum values. Categorical variables are expressed as number and percentage. Abbreviations: AISI, Aggregate Systemic Inflammation Index; BISAP, Bedside Index for Severity in Acute Pancreatitis; APACHE II, Acute Physiology and Chronic Health Evaluation II; HAPS, Harmless Acute Pancreatitis Score. Additional abbreviations are defined at the end of the manuscript.

**Table 2:** Frequency distribution table for blood parameter measurements.

Variable	Mean ± SD (Median)	Min.–Max.
Hemoglobin (g/dL)	13.41 ± 2.66 (13.40)	5.10–46.70
Platelet (×10^9^/L)	268.25 ± 89.75 (257.00)	80.00–697.00
Leukocyte (×10^9^/L)	10.59 ± 4.00 (9.80)	2.41–25.15
Creatinine (mg/dL)	0.99 ± 1.24 (0.77)	0.12–20.31
Albumin (g/L)	21.23 ± 18.52 (4.70)	1.90–51.00
CRP (g/L)	47.67 ± 66.97 (16.50)	0.50–400.00
BUN (mg/dL)	17.54 ± 11.92 (14.90)	3.70–92.93
Alanine Aminotransferase (U/L)	207.10 ± 221.94 (138.00)	6.00–1297.00
Total-bilirubin (mg/dL)	2.88 ± 3.36 (1.80)	0.20–25.80
Direct-bilirubin (mg/dL)	1.87 ± 2.61 (0.90)	0.00–19.10
Alkaline Phosphatase (U/L)	214.84 ± 191.26 (162.50)	23.00–1936.00
Gamma-glutamyl transferase (U/L)	343.17 ± 310.75 (276.50)	5.00–1545.00
Lipase (U/L)	1910.69 ± 1762.79 (1472.50)	20.00–12,917.00
Lactate dehydrogenase (U/L)	349.85 ± 289.05 (276.50)	123.00–4677.00
Aspartate Aminotransferase (U/L)	194.92 ± 256.20 (111.50)	6.00–2224.00
Neutrophil (×10^9^/L)	8.73 ± 4.04 (7.85)	1.79–24.75
Lymphocyte (×10^9^/L)	1.27 ± 0.66 (1.15)	0.18–5.03
Monocyte (×10^9^/L)	0.50 ± 0.18 (0.46)	0.06–1.30
Platelet (×10^9^/L)	265.72 ± 80.95 (254.00)	48.00–602.00
Amylase (U/L)	1239.70 ± 1092.89 (948.50)	22.00–7461.00
PLR	260.53 ± 145.99 (222.68)	26.93–890.91
MLR	0.48 ± 0.27 (0.41)	0.08–1.67
NLRP	0.04 ± 0.05 (0.03)	0.00–0.41
NLR	9.54 ± 8.51 (6.89)	0.78–57.15
SIRI	4.61 ± 4.28 (3.32)	0.33–4.28

Descriptive statistics of blood parameter measurements in patients diagnosed with acute pancreatitis. Continuous variables are summarized as mean ± standard deviation [median], accompanied by their corresponding minimum and maximum values. These descriptive analyses were used to characterize the overall distribution of hematologic, biochemical, inflammatory, and enzymatic markers within the study population. Additional abbreviations are defined in the List of Abbreviations at the end of the manuscript.

**Table 3:** Correlation and comparative analyses between laboratory parameters and clinical variables in patients with acute pancreatitis.

Laboratory Parameters	Length of Hospital Stay (Day)	Systemic Complication	Local Complication	Mortality	Biliary Pancreatitis	Sex (Male)	History of ERCP	Post-ERCP Pancreatitis	Age
Statistic	r/*p*	t/*p*	t/*p*	t/*p*	t/*p*	t/*p*	t/*p*	t/*p*	r/*p*
Alkaline Phosphatase (U/L)	0.179/0.001 *	−0.705/0.481	1.356/0.176	−0.439/0.661	−1.412/0.159	−0.362/0.717	−5.618/0.001 *	−2.529/0.001 *	0.125/0.030
Alanine Aminotransferase (U/L)	−0.146/0.001 *	−0.528/0.598	0.115/0.909	1.148/0.252	−5.471/0.001 *	2.994/0.003 *	1.020/0.308	2.412/0.017 *	0.155/0.002
Aspartate Aminotransferase (U/L)	0.051/0.030 *	−1.335/0.183	−0.142/0.887	0.111/0.911	−3.328/0.001 *	3.786/0.001 *	2.300/0.022 *	2.203/0.030 *	0.042/0.399
Albumin (g/L)	−0.037/0.459	−6.563/0.001 *	3.201/0.002 *	2.226/0.041 *	0.077/0.938	0.120/0.904	2.245/0.025 *	1.783/0.077	0.021 */0.669
Amylase (U/L)	0.152/0.001 *	−0.295/0.768	−0.281/0.779	0.057/0.955	−3.577/0.001 *	1.723/0.086	2.098/0.036 *	3.692/0.001 *	0.133/0.007
BUN (mg/dL)	0.237/0.001 *	−3.944/0.001 *	−0.603/0.547	−3.335/0.005 *	0.078/0.938	−0.058/0.977	0.911/0.322	1.311/0.191	0.386/0.001
CRP (g/L)	0.212/0.001 *	−5.023/0.001 *	−2.249/0.027 *	−1.052/0.294	−0.073/0.942	−2.925/0.001 *	0.886/0.376	2.531/0.012 *	0.117/0.017
Creatinine (mg/dL)	0.130/0.008 *	−2.143/0.034 *	0.891/0.373	−2.128/0.051	0.792/0.429	−1.050/0.294	0.171/0.865	0.113/0.910	0.179/0.001
Direct bilirubin (mg/dL)	0.187/0.001 *	−0.393/0.695	2.654/0.009 *	−0.493/0.622	−3.121/0.001 *	−1.312/0.190	−5.001/0.001 *	−1.975/0.051	0.107/0.030
Gamma-glutamyl transferase (U/L)	−0.005/0.927	−0.203/0.839	0.953/0.341	−0.260/0.795	−5.429/0.001 *	−0.104/0.917	−3.729/0.001 *	−0.830/0.407	0.030 */0.543
Hemoglobin (g/dL)	0.151/0.002 *	1.865/0.063	−1.429/0.154	2.576/0.010 *	0.608/0.544	−5.018/0.001 *	0.770/0.442	0.348/0.728	0.247/0.001
Lactate dehydrogenase (U/L)	0.143/0.001 *	−2.592/0.001 *	1.105/0.270	−0.219/0.827	−2.262/0.024 *	3.721/0.001 *	3.209/0.001 *	3.893/0.001 *	0.042 */0.397
Leukocyte (×10^9^/L)	0.084/0.089	−5.481/0.001 *	−2.068/0.041 *	−0.054/0.957	−0.164/0.870	−2.097/0.037 *	2.458/0.014 *	4.013/0.001 *	0.012 */0.807
Lipase (U/L)	0.139/0.005	0.539/0.590	−0.009/0.993	0.369/0.712	−2.477/0.014 *	1.751/0.081	1.488/0.137	0.627/0.531	0.156/0.002
Lymphocyte (×10^9^/L)	−0.139/0.005 *	4.104/0.001 *	0.829/0.408	2.852/0.005 *	3.684/0.001 *	−0.924/0.356	1.848/0.065	−0.174/0.862	0.241/0.001
Monocyte (×10^9^/L)	−0.073/0.137	−0.977/0.330	−0.859/0.392	0.318/0.755	−0.824/0.411	−3.836/0.001 *	1.084/0.279	2.336/0.021 *	0.068/0.167
Neutrophil (×10^9^/L)	0.116/0.018 *	−6.492/0.001 *	−2.256/0.025 *	−1.553/0.142	−0.680/0.497	−1.699/0.090	2.627/0.009 *	3.826/0.001 *	0.042 */0.391
Platelet	0.004/0.930	−0.421/0.674	−0.230/0.818	2.094/0.037 *	0.703/0.483	3.683/0.001 *	0.881/0.379	−0.432/0.666	0.140/0.004
Total-bilirubin (mg/dL)	0.171/0.001 *	−0.538/0.591	2.566/0.011 *	−0.517/0.605	−3.443/0.001 *	−1.295/0.196	−4.421/0.001 *	−1.398/0.165	0.095/0.053

Pearson’s correlation was applied to assess relationships with continuous variables, while group comparisons for binary variables were performed using independent sample *t*-tests. Correlation coefficients (r), t statistics (t), and *p*-values (*p*) are reported. A *p*-value < 0.05 was considered statistically significant. Statistically significant results are indicated with an asterisk (*). Abbreviations are defined in the List of Abbreviations at the end of the manuscript.

**Table 4:** Correlations between laboratory parameters and clinical scoring systems in patients with acute pancreatitis.

Parameter	Glasgow 2	Marshall	BISAP	RANSON	Apache II	HAPS
Statistic	r/*p*	r/*p*	r/*p*	r/*p*	r/*p*	r/*p*
Alkaline Phosphatase (U/L)	0.134/0.001 *	0.009/0.861	0.060/0.221	0.108/0.028 *	0.052/0.296	0.152/0.001 *
Alanine Aminotransferase (U/L)	0.122/0.013 *	0.039/0.428	0.147/0.001 *	0.237/0.001 *	0.165/0.001 *	0.106/0.032 *
Aspartate Aminotransferase (U/L	0.041/0.412	0.057/0.247	0.026/0.603	0.361/0.001 *	−0.009/0.853	0.044/0.368
Albumin (g/L)	0.019/0.695	0.021/0.676	0.036/0.465	0.017/0.723	0.135/0.006 *	0.139/0.005 *
Amylase (U/L)	0.115/0.020 *	0.041/0.404	0.156/0.001 *	0.134/0.001 *	−0.134/0.001 *	0.011/0.082
BUN (mg/dL)	0.484/0.001 *	0.480/0.001 *	0.546/0.001 *	0.237/0.001 *	0.625/0.001 *	0.048/0.331
CRP (g/L)	0.327/0.001 *	0.207/0.001 *	0.349/0.001 *	0.213/0.001 *	0.301/0.001 *	0.089/0.072
Creatinine (mg/dL)	0.315/0.001 *	0.611/0.001 *	0.349/0.001 *	0.165/0.001 *	0.530/0.001 *	0.081/0.102
Direct-bilirubin (mg/dL)	0.124/0.012 *	0.017/0.730	0.091/0.065	0.089/0.072	0.072/0.143	0.027/0.584
Gamma-glutamyl transferase (U/L)	0.003/0.946	0.029/0.555	0.049/0.321	0.102/0.038 *	−0.032/0.513	0.083/0.093
Hemoglobin (g/dL)	0.259/0.001 *	0.177/0.001 *	0.295/0.001 *	0.087/0.079	0.314/0.001 *	0.324/0.001 *
Lactate dehydrogenase (U/L)	0.199/0.001 *	0.298/0.001 *	0.159/0.001 *	0.406/0.001 *	0.208/0.001 *	0.119/0.016 *
Leukocyte (×10^9^/L)	0.221/0.001 *	0.056/0.259	0.179/0.001 *	0.262/0.001 *	0.112/0.023 *	0.180/0.001 *
Lipase (U/L)	0.145/0.003 *	0.001/0.989	0.173/0.001 *	0.076/0.126	−0.179/0.001 *	0.050 */0.312
Lymphocyte (×10^9^/L)	0.189/0.001 *	0.123/0.012 *	0.241/0.001 *	0.277/0.001 *	−0.255/0.001 *	0.021/0.672
Monocyte (×10^9^/L)	0.123/0.012 *	0.058/0.243	0.045/0.366	0.160/0.001 *	0.053/0.286	0.056/0.261
Neutrophil (×10^9^/L)	0.284/0.001 *	0.114/0.021 *	0.255/0.001 *	0.295/0.001 *	0.206/0.001 *	0.145/0.003 *
Platelet	0.148/0.003 *	0.070/0.158	0.170/0.001 *	0.036/0.465	−0.139/0.005 *	0.032/0.512
Total-bilirubin (mg/dL)	0.100/0.001 *	0.017/0.738	0.092/0.063	0.090/0.066	0.063/0.201	0.007/0.894

Correlations between laboratory parameters and clinical scoring systems were assessed using the Pearson correlation test. Correlation coefficients (r) represent the strength and direction of linear relationships between continuous variables. Statistical significance was set at *p* < 0.05, and significant correlations are marked with an asterisk (*). Parameters without statistically significant associations are not emphasized. Abbreviations are defined in the List of Abbreviations at the end of the manuscript.

**Table 5:** Correlation and group comparison of inflammatory indices with clinical outcomes in acute pancreatitis.

A. Correlation Coefficients (r) for Continuous Variables						
**Variable**	**PLR**	**NLRP**	**NLR**	**AISI**	**SIRI**	**Modified AISI**
**Statistic**	**r/*p***	**r/*p***	**r/*p***	**r/*p***	**r/*p***	**r/*p***
Hospital Stay	0.122/0.013 *	0.141/0.004 *	0.157/0.001 *	0.220/0.001 *	0.239/0.001 *	0.138/0.005 *
Glasgow 2	0.158/0.001 *	0.316/0.001 *	0.308/0.001 *	0.315/0.001 *	0.365/0.001 *	0.237/0.001 *
Marshall	0.095/0.055	0.149/0.001 *	0.168/0.001 *	0.160/0.001 *	0.155/0.001 *	0.131/0.008 *
BISAP	0.243/0.001 *	0.430/0.001 *	0.434/0.001 *	0.283/0.001 *	0.368/0.001 *	0.204/0.001 *
RANSON	0.291/0.001 *	0.271/0.001 *	0.351/0.001 *	0.415/0.001 *	0.403/0.001 *	0.503/0.001 *
APACHE II	0.179/0.001 *	0.290/0.001 *	0.312/0.001 *	0.272/0.001 *	0.303/0.001 *	0.165/0.001 *
HAPS Score	−0.004/0.939	0.020/0.679	0.067/0.176	0.117/0.017 *	0.092/0.061	0.174/0.001 *
Age	0.232/0.001 *	0.272/0.001 *	0.280/0.001 *	0.135/0.001 *	0.181/0.001 *	0.100/0.042 *
B. Independent Sample t-Test Results for Binary Variables						
**Group Comparison**	**PLR**	**NLRP**	**NLR**	**AISI**	**SIRI**	**Modified AISI**
**Statistic**	**t/*p***	**t/*p***	**t/*p***	**t/*p***	**t/*p***	**t/*p***
Systemic Complication	−4.777/0.001 *	−5.191/0.001 *	−6.384/0.001 *	−6.214/0.001 *	−6.257/0.001 *	−5.457/0.001 *
Local Complication	−1.252/0.191	−1.722/0.088	−1.932/0.083	−2.254/0.025 *	−2.025/0.047 *	−1.783/0.078
Mortality	−0.856/0.393	−2.824/0.013 *	−2.691/0.017 *	−1.810/0.091	−2.038/0.043 *	−1.858/0.145
Biliary Pancreatitis	−0.868/0.004 *	−1.940/0.053	−2.812/0.005 *	−2.762/0.006 *	−3.066/0.002 *	−3.628/0.001 *
Sex (Female vs. Male)	−2.352/0.019 *	−1.261/0.208	−0.639/0.523	−1.152/0.250	−2.120/0.033 *	−0.100/0.920
ERCP History	−1.237/0.217	−0.012/0.990	−0.115/0.909	0.412/0.680	0.121/0.904	1.344/0.180
Post-ERCP Pancreatitis	0.288/0.773	2.497/0.013 *	−3.904/0.001 *	−3.797/0.001 *	4.910/0.001 *	6.878/0.001 *

This table includes two parts evaluating the associations between inflammatory indices and clinical outcomes in patients with acute pancreatitis. Table 5A presents the Pearson correlation coefficients (r) for continuous variables, including age, length of hospital stay, and clinical severity scores (e.g., Glasgow, Ranson, APACHE II). Positive r-values indicate a direct association, while negative r-values reflect an inverse relationship. Table 5B provides the results of independent sample *t*-tests (t), comparing inflammatory indices across binary clinical outcomes (e.g., presence or absence of complications, mortality, sex, post-ERCP status). Statistical significance was defined as *p* < 0.05 and * *p* < 0.01. Abbreviations are defined in the List of Abbreviations at the end of the manuscript.

**Table 6:** Comparison of systemic inflammation indices across acute pancreatitis severity levels as defined by the revised Atlanta classification (2012).

Inflammatory Index	Severity (Group)	Mean	SD	ANOVA (F)	*p*-Value	Post hoc (Tukey HSD)
PLR	Mild (1)	236.91	125.70	13.145	0.001 *	3 > 1, 3 > 2, 2 > 1
	Moderate (2)	273.38	156.11			
	Severe (3)	354.90	178.20			
MLR	Mild (1)	0.43	0.24	15.297	0.001 *	3 > 1, 3 > 2, 2 > 1
	Moderate (2)	0.52	0.27			
	Severe (3)	0.66	0.35			
NLRP	Mild (1)	0.03	0.03	19.056	0.001 *	3 > 1, 3 > 2, 2 > 1
	Moderate (2)	0.05	0.06			
	Severe (3)	0.07	0.08			
NLR	Mild (1)	7.29	5.59	25.082	0.001 *	3 > 1, 3 > 2, 2 > 1
	Moderate (2)	11.86	10.23			
	Severe (3)	15.17	11.58			
AISI	Mild (1)	920.21	816.70	26.861	0.001 *	3 > 1, 3 > 2, 2 > 1
	Moderate (2)	1382.59	1055.16			
	Severe (3)	2064.64	1641.24			
Modified AISI	Mild (1)	1538.16	2034.23	23.338	0.001 *	3 > 1, 3 > 2, 2 > 1
	Moderate (2)	2354.62	2352.49			
	Severe (3)	4261.11	4573.60			
SIRI	Mild (1)	3.51	3.00	26.804	0.001 *	3 > 1, 3 > 2, 2 > 1
	Moderate (2)	5.53	4.34			
	Severe (3)	7.97	7.07			

Data are presented as mean ± standard deviation (SD). Comparisons between the three severity groups—mild (Group 1), moderate (Group 2), and severe (Group 3)—were performed using one-way analysis of variance (ANOVA), followed by Tukey’s Honest Significant Difference (HSD) test for post hoc analysis. Severity classification is based on the revised Atlanta criteria (2012). A *p*-value < 0.05 was considered statistically significant (*). Abbreviations are defined in the List of Abbreviations at the end of the manuscript.

**Table 7:** ROC analysis results.

Variable	Category	Predictive Value	Sensitivity	1-Spesificity	Positive Value	Negative Value	Odds Ratio
	(Reference)						(CI)
Hospitalization Time (Days)	Under 10 Days (r)	236.626 *	94.40%	91.00%	65.05%	34.95%	0.385
	Over 10 Days						(0.326–0.444)
Ranson Situation	0 (r)	113.189 *	100%	98.80%	18.45%	81.55%	0.295
	1+						(0.240–0.351)
Ranson Situation	0 (r)	99.287 **	100%	99.70%	18.45%	81.55%	0.232
	1+						(0.183–0.281)

(r) = Reference Category; * AISI Level; ** Modified AISI Level; CI: Confidence Interval. Abbreviations are defined in the List of Abbreviations at the end of the manuscript.

## Data Availability

The original contributions presented in this study are included in the article. Further inquiries can be directed to the corresponding author.

## Abbreviations

AISI	Aggregate Systemic Inflammation Index
ALT	Alanine Aminotransferase
AP	Acute Pancreatitis
APACHE II	Acute Physiology and Chronic Health Evaluation II
AST	Aspartate Aminotransferase
BISAP	Bedside Index for Severity in Acute Pancreatitis
BUN	Blood Urea Nitrogen
CBC	Complete Blood Count
CI	Confidence Interval
CRP	C-Reactive Protein
CT	Computed Tomography
ERCP	Endoscopic Retrograde Cholangiopancreatography
ERCP History	ERCP was performed during hospitalization
GGT	Gamma-Glutamyl Transferase
HAPS	Harmless Acute Pancreatitis Score
IL	Interleukin
LDH	Lactate Dehydrogenase
LMR	Lymphocyte-to-Monocyte Ratio
LOS	Length of Stay
MRI	Magnetic Resonance Imaging
NLR	Neutrophil-to-Lymphocyte Ratio
NLRP	Neutrophil-to-Lymphocyte*Platelet Ratio
PLR	Platelet-to-Lymphocyte Ratio
PCT	Procalcitonin
PPV	Positive Predictive Value
RDW	Red Cell Distribution Width
ROC	Receiver Operating Characteristic
SD	Standard Deviation
SIRI	Systemic Inflammatory Response Index
SIRS	Systemic Inflammatory Response Syndrome
TNF-α	Tumor Necrosis Factor-alpha
WBC	White Blood Cell
